# Molecular Phylogeny and Biogeography of the Amphidromous Fish Genus *Dormitator* Gill 1861 (Teleostei: Eleotridae)

**DOI:** 10.1371/journal.pone.0153538

**Published:** 2016-04-13

**Authors:** Sesángari Galván-Quesada, Ignacio Doadrio, Fernando Alda, Anabel Perdices, Ruth Gisela Reina, Martín García Varela, Natividad Hernández, Antonio Campos Mendoza, Eldredge Bermingham, Omar Domínguez-Domínguez

**Affiliations:** 1 Programa Institucional de Doctorado en Ciencias Biológicas, Universidad Michoacana de San Nicolás de Hidalgo, Morelia, Michoacán, México; 2 Laboratorio de Biología Acuática, Facultad de Biología, Universidad Michoacana de San Nicolás de Hidalgo, Morelia, Michoacán, México; 3 Departamento de Biodiversidad y Biología Evolutiva, Museo Nacional de Ciencias Naturales, CSIC, Madrid, Spain; 4 Smithsonian Tropical Research Institute, Apartado 2072, Balboa, Republic of Panama; 5 Instituto de Biología, Universidad Nacional Autónoma de México, Distrito Federal, México; 6 Instituto de Medicina Tropical Pedro Kourí, Apartado 601, La Habana, Cuba; University of California Santa Cruz, UNITED STATES

## Abstract

Species of the genus *Dormitator*, also known as sleepers, are representatives of the amphidromous freshwater fish fauna that inhabit the tropical and subtropical coastal environments of the Americas and Western Africa. Because of the distribution of this genus, it could be hypothesized that the evolutionary patterns in this genus, including a pair of geminate species across the Central American Isthmus, could be explained by vicariance following the break-up of Gondwana. However, the evolutionary history of this group has not been evaluated. We constructed a time-scaled molecular phylogeny of *Dormitator* using mitochondrial (Cytochrome *b*) and nuclear (Rhodopsin and β-actin) DNA sequence data to infer and date the cladogenetic events that drove the diversification of the genus and to relate them to the biogeographical history of Central America. Two divergent lineages of *Dormitator* were recovered: one that included all of the Pacific samples and another that included all of the eastern and western Atlantic samples. In contrast to the Pacific lineage, which showed no phylogeographic structure, the Atlantic lineage was geographically structured into four clades: Cameroon, Gulf of Mexico, West Cuba and Caribbean, showing evidence of potential cryptic species. The separation of the Pacific and Atlantic lineages was estimated to have occurred ~1 million years ago (Mya), whereas the four Atlantic clades showed mean times of divergence between 0.2 and 0.4 Mya. The splitting times of *Dormitator* between ocean basins are similar to those estimated for other geminate species pairs with shoreline estuarine preferences, which may indicate that the common evolutionary histories of the different clades are the result of isolation events associated with the closure of the Central American Isthmus and the subsequent climatic and oceanographic changes.

## Introduction

Fish display a wide array of life-history strategies, many of which affect their dispersal potential and their subsequent geographic differentiation and intraspecific variability [1–3]. The implications and differences in ecology, evolutionary history and biogeography associated with different life-history strategies have been the focus of scientific debate from the time of Darwin [4] until the present [5–8]. For example, diadromy is a life-history behavior in which individuals spend predictable phases of their life cycle in freshwater rivers or in the ocean, typically for feeding or reproduction [9]. Because of their intermediate relationships with the continental and marine realms, fishes with this life-history strategy are usually characterized by high dispersal potential, widespread distributions and shallow genetic differentiation [5,10].

Brackish environments in the Neotropics host a substantial amount of diadromous fish fauna, including gobies, mullets, snooks and sleepers. However, few studies have been conducted on these groups of fish, which represent an important component of the biodiversity of these ecosystems [11]. Investigations of the evolutionary history of diadromous taxa are even rarer despite the value of studying their genetic variation for understanding the evolution of coastal environments and its influence on species geographic distribution [12]. For example, a recent study of *Agonostomus monticola* highlighted the importance of major vicariant events in generating cryptic diversity in diadromous species [10].

In the Neotropics, the closing of the Central American Seaway represented a dramatic event separating marine and coastal organisms and facilitating the emergence of geminate species pairs on each side of the Isthmus that followed independent evolutionary trajectories [13]. However, comparisons of divergence times among geminate species pairs do not support a single and simultaneous divergence time for all taxa, suggesting that species might have responded differently to the complex geological evolution of the Isthmus and its new habitat development [14–16]. For instance, geminate pairs of gastropods inhabiting high intertidal mangroves show shallower genetic divergences compared to those inhabiting lower intertidal and subtidal environments [17]. Hence, ecological differences, such as habitat depth, may influence the timing of separation between geminate clades [17,18].

Fish species of the genus *Dormitator* Gill 1861 (Teleostei: Eleotridae) are amphidromous, which is a form of diadromy in which adults live and reproduce in freshwaters. After the eggs hatch, the larvae of this kind of species drift to the sea, where they spend a variable amount of time, potentially dispersing long distances, before returning to freshwater streams [19]. Their common name “sleepers” arises from the apparent lack of motility of these species as adults [20]. *Dormitator* inhabit freshwater and brackish environments along the tropical and subtropical coasts and estuaries of the eastern Pacific and Atlantic Oceans [21,22]. On the Pacific coast, the genus is distributed from the Gulf of California to Peru [22], including the Galapagos Islands [23]. On the western Atlantic coast, the distribution area ranges from North Carolina to Brazil [22], including the Antilles. On the eastern Atlantic coast, the genus ranges from Senegal to Angola [24]. Four species are recognized in the Atlantic [24–27]: *Dormitator maculatus* (Bloch 1792), which is found in the western Atlantic from southern USA to Central America and southeastern Brazil; *Dormitator cubanus* Ginsburg 1953 in western Cuba (Pinar del Río); *Dormitator lophocephalus* Hoedeman 1951 in Suriname; and *Dormitator lebretonis* (Steindachner 1870) occurring from Senegal to Namibia. In contrast, only one species is found on the eastern Pacific coast: *Dormitator latifrons* (Richardson 1844). Within this region, *Dormitator latifrons mexicanus* Ginsburg 1953, restricted to the Pacific coast of Mexico, has been described as a distinct subspecies from the nominal *Dormitator latifrons latifrons*.

Probably because of its wide distribution range, *Dormitator* species have suffered from considerable taxonomic instability, including the use of at least 20 synonyms [24–27]. However, *Dormitator* have been the subject of few systematic studies [28–30], and their genetic diversity and phylogenetic relationships are unknown. Furthermore, because of the species distribution on both sides of the Atlantic and their allopatric distribution on the Pacific and the Atlantic slopes of the Americas, it could be hypothesized that the splitting of continentally disjunct lineages of *Dormitator* coincides with the break-up of Gondwana in the Early Cretaceous and that this genus may include at least one pair of geminate species: *D*. *latifrons* and *D*. *maculatus* [13,31]. The genus *Dormitator* is therefore a good model to 1) study the effects of geological events and life-history strategies (amphidromy) on species divergence and distribution patterns and 2) uncover hidden diversity of putative cryptic and geminate species pairs by using molecular methods. To address these objectives, we inferred a molecular (mitochondrial and nuclear) phylogenetic hypothesis of *Dormitator* species and used molecular clock analyses to investigate the patterns and timescale of lineage divergence across its distribution in America and Africa.

## Materials and Methods

### Ethics statement

Field collections did not involve endangered or protected species. Field and laboratory protocols used in this study, including sampling procedures, were reviewed and approved by the Mexican Ministry of Environmental and Natural Resources (SEMARNAT), under collection permit number FAUT 0202. Further approval by an ethics committee was not necessary because this research did not include animal experimentation. Samples requested from other institutions, such as the Smithsonian Tropical Research Institute Neotropical Fish Collection (STRI, Panama) and Museo Nacional de Ciencias Naturales (CSIC, Spain), were also used. These samples were collected following sampling procedures reviewed and approved by STRI’s “Institutional Animal Care and Use Committee” and by CSIC’s “Ethics Committee”, respectively.

### Specimen collection

Specimens were captured with seine, gill nets or electrofishing when possible. Immediately after capture, all individuals were anesthetized using Tricaine methanesulfonate (MS-222) to alleviate suffering. Tissue samples (~3 mm^2^ fin clips) were obtained, preserved in 95% ethanol or DMSO buffer and stored at 4°C. Once tissue samples were collected, fish were released at the same collecting site after confirming recovery of total motility (n = 100), or fish were humanely euthanized with an overdose of MS-222 (n = 158). Death was confirmed after no gill movement was observed for at least 10 minutes. Specimens were then individually tagged, fixed in formalin and then transferred to 70% ethanol for long-term storage. Voucher specimens are deposited in the Colección Ictiológica de la Facultad de Biología (CPUM-Universidad Michoacana de San Nicolás de Hidalgo, Mexico), Museo Nacional de Ciencias Naturales (CSIC Spain), Smithsonian Tropical Research Institute Neotropical Fish Collection (Panama) and Colección Nacional de Peces, Instituto de Biología (Universidad Nacional Autónoma de México, Mexico).

**Fig 1:**
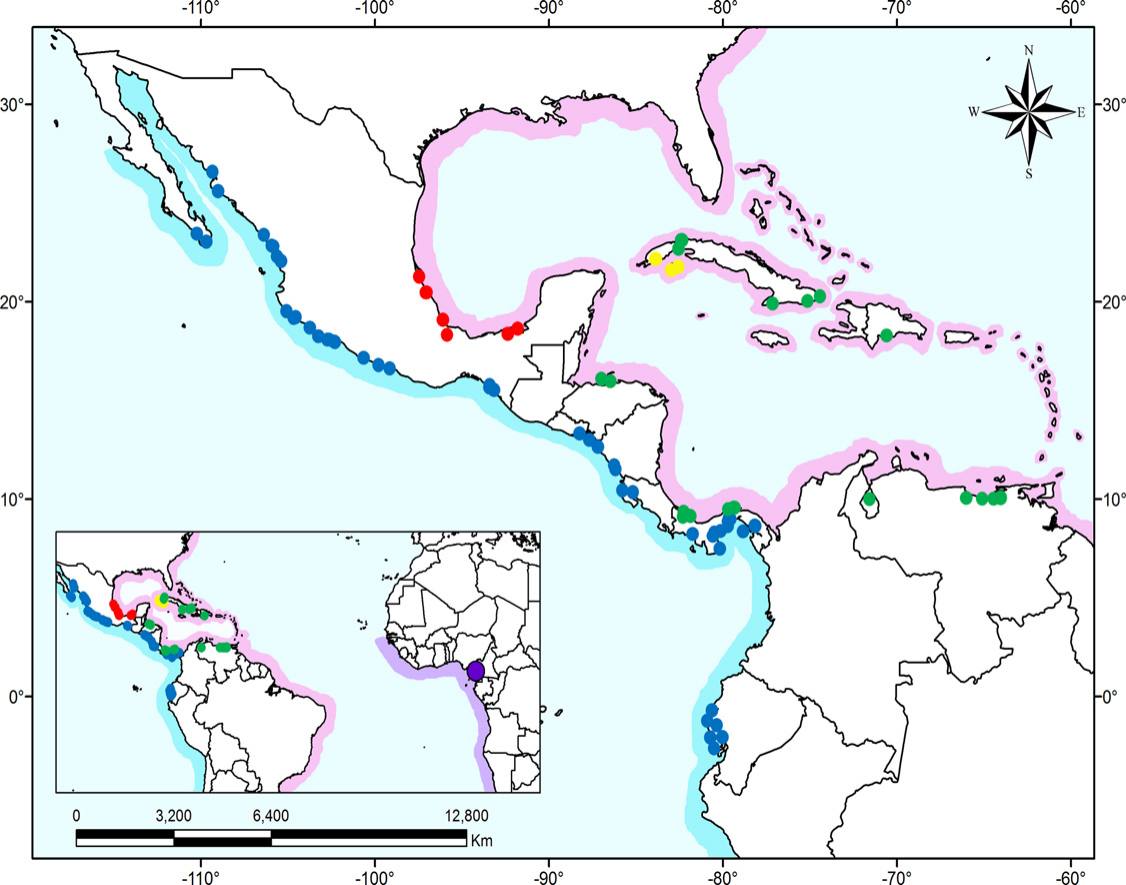
Distribution of *Dormitator* species and sampling locations. Shaded areas represent the distribution range of *Dormitator*. Blue shading indicates the Pacific distribution (*D*. *latifrons*), pink shading indicates the western Atlantic distribution (including *D*. *maculatus*, *D*. *cubanus* and *D*. *lophocephalus*), and purple shading indicates the eastern Atlantic distribution areas (*D*. *lebretonis*). Dots represent sampling locations, and the colors represent the monophyletic groups recovered by our phylogenetic hypothesis as detailed below. Dot color code: blue = Pacific; red = Gulf of Mexico; purple = Cameroon; green = Caribbean; and yellow = West Cuba.

*Dormitator* specimens were collected from coastal environments along the Pacific and Atlantic slopes of the Americas and the eastern Atlantic coast of Africa. According to the described species distribution, our sampling, comprising 84 collecting locations, included specimens of *D*. *latifrons* (both putative subspecies), *D*. *maculatus*, *D*. *cubanus* and *D*. *lebretonis* (Fig 1, Table 1). Additionally, samples of *Gobiomorus dormitor* and *Eleotris senegalensis* were included in the analyses as outgroups because of their close evolutionary relationship with *Dormitator* [28–30].

**Table 1:** Summary of *Dormitator* samples and locations analyzed in this study.

Location No.	Species	Location Name	Location Code	Country	Oceanic slope	No. of Cyt*b* sequences (GenBank accession numbers: KU764789-KU765046)	No. of Rh sequences (GenBank accession numbers: KU765049-KU765129)	No. of β-actin sequences (GenBank accession numbers: KU958384-KU958464)	Clade (Concatenated data set)
1	*D*. *latifrons*	Estero La Poza, Todos Santos, Baja California Sur	BCS1MEX	Mexico	Pacific	4	1	1	Pacific
2	*D*. *latifrons*	Oasis San José del Cabo, Baja California Sur	BCS2MEX	Mexico	Pacific	3	1	1	Pacific
3	*D*. *latifrons*	Río Las Bocas, Sonora	Son1MEX	Mexico	Pacific	1	1	1	Pacific
4	*D*. *latifrons*	Arroyo El Guajare, Sonora	Son2MEX	Mexico	Pacific	3	2	2	Pacific
5	*D*. *latifrons*	Topolobampo, Sinaloa	Sin1MEX	Mexico	Pacific	4	1	1	Pacific
6	*D*. *latifrons*	Laguna Las Cañas, Escuinapa, Sinaloa	Sin2MEX	Mexico	Pacific	3	2	2	Pacific
7	*D*. *latifrons*	Poza, Escuinapa, Sinaloa	Sin3MEX	Mexico	Pacific	3	2	2	Pacific
8	*D*. *latifrons*	Laguna El Huizache, Walamo, Sinaloa	Sin4MEX	Mexico	Pacific	3	2	2	Pacific
9	*D*. *latifrons*	San Pedro, Nayarit	NayMEX	Mexico	Pacific	4	2	2	Pacific
10	*D*. *latifrons*	Barra de Navidad, Jalisco	Jal1MEX	Mexico	Pacific	3	2	2	Pacific
11	*D*. *latifrons*	Arroyo Seco, Jalisco	Jal2MEX	Mexico	Pacific	4	2	2	Pacific
12	*D*. *latifrons*	Estero Chamela, Jalisco	Jal3MEX	Mexico	Pacific	4	1	1	Pacific
13	*D*. *latifrons*	Boca de Apiza, Colima	ColMEX	Mexico	Pacific	2	2	2	Pacific
14	*D*. *latifrons*	Barra de Pichi, Michoacán	Mich1MEX	Mexico	Pacific	14	3	3	Pacific
15	*D*. *latifrons*	Cachán, Michoacán	Mich3MEX	Mexico	Pacific	2	1	1	Pacific
16	*D*. *latifrons*	Estero Teolán, Michoacán	Mich4MEX	Mexico	Pacific	4	2	2	Pacific
17	*D*. *latifrons*	Estero Santa Ana, Michoacán	Mich6MEX	Mexico	Pacific	2	2	2	Pacific
18	*D*. *latifrons*	Estero Mexcalhuacan, Michoacán	Mich7MEX	Mexico	Pacific	1	1	1	Pacific
19	*D*. *latifrons*	El Tamarindo Chautengo, Guerrero	Gro1MEX	Mexico	Pacific	3	1	1	Pacific
20	*D*. *latifrons*	Laguna Tres Palos, Guerrero	Gro2MEX	Mexico	Pacific	4	2	2	Pacific
21	*D*. *latifrons*	La Vinata, Guerrero	Gro3MEX	Mexico	Pacific	6	2	2	Pacific
22	*D*. *latifrons*	Rión Pijijiapan, Chiapas	Chis1MEX	Mexico	Pacific	4	0	0	Pacific
23	*D*. *latifrons*	La Conquista, Chiapas	Chis2MEX	Mexico	Pacific	5	0	0	Pacific
24	*D*. *latifrons*	Joaquín Amaro, Pijijiapan, Chiapas	Chis3MEX	Mexico	Pacific	4	0	0	Pacific
25	*D*. *latifrons*	El Tránsito cerca de San Miguel	TranSA	El Salvador	Pacific	3	0	0	Pacific
26	*D*. *latifrons*	Barranco, Río Escalante	BarrNI	Nicaragua	Pacific	2	1	1	Pacific
27	*D*. *latifrons*	El Viejo, Río Atoya	AtoyNI	Nicaragua	Pacific	4	2	2	Pacific
28	*D*. *latifrons*	Israel, Río La Chepa	ChepNI	Nicaragua	Pacific	1	1	1	Pacific
29	*D*. *latifrons*	Río Escalante	EscaNI	Nicaragua	Pacific	2	0	0	Pacific
30	*D*. *latifrons*	Guanacaste	GuanCR	Costa Rica	Pacific	1	0	0	Pacific
31	*D*. *latifrons*	Río Canas	CanaCR	Costa Rica	Pacific	2	0	0	Pacific
32	*D*. *latifrons*	Puerto Caimito	PrtoPA	Panama	Pacific	1	0	0	Pacific
33	*D*. *latifrons*	Río Chico-Río Grande	ChicPA	Panama	Pacific	10	0	0	Pacific
34	*D*. *latifrons*	Río Farallon	FaraPA	Panama	Pacific	2	0	0	Pacific
35	*D*. *latifrons*	Río Santa María oxbow	MariPA	Panama	Pacific	2	0	0	Pacific
36	*D*. *latifrons*	Río El Pajonal	PajoPA	Panama	Pacific	1	0	0	Pacific
37	*D*. *latifrons*	Río Grande Empire Range pools above Bridge	GranPA	Panama	Pacific	2	0	0	Pacific
38	*D*. *latifrons*	Punta Chame outer beach and inside lagoon	ChamPA	Panama	Pacific	2	2	2	Pacific
39	*D*. *latifrons*	Río Zapallal	ZapaPA	Panama	Pacific	1	0	0	Pacific
40	*D*. *latifrons*	Station near Santa Fe, Darien	DariPA	Panama	Pacific	1	0	0	Pacific
41	*D*. *latifrons*	Río Cardenas	Car1PA	Panama	Pacific	4	0	0	Pacific
42	*D*. *latifrons*	Isla del Rey	ReyPA	Panama	Pacific	1	0	0	Pacific
43	*D*. *latifrons*	Río Cardenas 2	Car2PA	Panama	Pacific	1	0	0	Pacific
44	*D*. *latifrons*	Río Caimito	CaimPA	Panama	Pacific	1	0	0	Pacific
45	*D*. *latifrons*	Río Santiago	SantPA	Panama	Pacific	1	0	0	Pacific
46	*D*. *latifrons*	Río Cardenas 3	Car3PA	Panama	Pacific	2	0	0	Pacific
47	*D*. *latifrons*	Puerto Cayo	CayoEC	Ecuador	Pacific	2	1	1	Pacific
48	*D*. *latifrons*	Estero Puerto López	LopeEC	Ecuador	Pacific	2	0	0	Pacific
49	*D*. *latifrons*	Yampe	YampEC	Ecuador	Pacific	2	2	2	Pacific
50	*D*. *latifrons*	Dos Mangas del pozo	MangEC	Ecuador	Pacific	3	0	0	Pacific
51	*D*. *latifrons*	Playa Guayas	GuayEC	Ecuador	Pacific	3	0	0	Pacific
52	*D*. *latifrons*	Barrio San Vicente	ViceEC	Ecuador	Pacific	2	1	1	Pacific
53	*D*. *maculatus*	Laguna de Tamiahua, Veracruz	Ver1MEX	Mexico	Atlantic	10	2	2	Gulf of Mexico
54	*D*. *maculatus*	Tecolutla, Veracruz	Ver2MEX	Mexico	Atlantic	10	3	3	Gulf of Mexico
55	*D*. *maculatus*	Tlacotalpan, Veracruz	Ver3MEX	Mexico	Atlantic	9	3	3	Gulf of Mexico
56	*D*. *maculatus*	Puente Las Cañas, Veracruz	Ver4MEX	Mexico	Atlantic	5	2	2	Gulf of Mexico
57	*D*. *maculatus*	Arroyo Moreno, Boca del Río, Veracruz	Ver5MEX	Mexico	Atlantic	7	2	2	Gulf of Mexico
58	*D*. *maculatus*	El Ancla, Campeche	CampMEX	Mexico	Atlantic	3	2	2	Gulf of Mexico
59	*D*. *maculatus*	Puente Guao, Tabasco	TabMEX	Mexico	Atlantic	1	0	0	Gulf of Mexico
60	*D*. *cubanus*	La Fe, Isla de la Juventud	LaFeCU	Cuba	Atlantic	3	1	1	West Cuba
61	*D*. *cubanus*	Mella, Isla de la Juventud	MellCU	Cuba	Atlantic	2	2	2	West Cuba
62	*D*. *cubanus*	La Reforma, Isla de la Juventud	RefoCU	Cuba	Atlantic	2	2	2	West Cuba
63	*D*. *cubanus*	Boca de Galafre, Pinar del Río	GalaCU	Cuba	Atlantic	2	1	1	West Cuba
64	*D*. *maculatus*	Guajaiton	GuajCU	Cuba	Atlantic	1	0	0	Caribbean
65	*D*. *maculatus*	Guanabo	GuanCU	Cuba	Atlantic	3	0	0	Caribbean
66	*D*. *maculatus*	Mota Dos, Gramma	MotaCU	Cuba	Atlantic	1	0	0	Caribbean
67	*D*. *maculatus*	Paraguay, Guantánamo	ParaCU	Cuba	Atlantic	2	1	1	Caribbean
68	*D*. *maculatus*	Ojo de Agua, Baracoa, Guantánamo	OjoCU	Cuba	Atlantic	4	3	3	Caribbean
69	*D*. *maculatus*	La Habana	HabaCU	Cuba	Atlantic	5	3	3	Caribbean
70	*D*. *maculatus*	República Dominicana	DR	Dominican Republic	Atlantic	3	0	0	Caribbean
71	*D*. *maculatus*	Jicotea Pond	JicoHO	Honduras	Atlantic	3	0	0	Caribbean
72	*D*. *maculatus*	Cayo Cochino	CayoHO	Honduras	Atlantic	2	0	0	Caribbean
73	*D*. *maculatus*	Río Cuanche	CuanPA	Panama	Atlantic	9	1	1	Caribbean
74	*D*. *maculatus*	Laguna de Chiriquí, Quebrada Larga	ChirPA	Panama	Atlantic	1	0	0	Caribbean
75	*D*. *maculatus*	Quebrada San Juan Río Cuango	JuanPA	Panama	Atlantic	3	1	1	Caribbean
76	*D*. *maculatus*	Quebrada on Km34 at Punta Peña-Road to Almirante	PenaPA	Panama	Atlantic	1	0	0	Caribbean
77	*D*. *maculatus*	2 quebradas before Big Creek—Isla Colón	ColoPA	Panama	Atlantic	1	0	0	Caribbean
78	*D*. *maculatus*	Río Cuango	CuangPA	Panama	Atlantic	1	0	0	Caribbean
79	*D*. *maculatus*	Barcelona-Playa Lido	LidoVE	Venezuela	Atlantic	2	2	2	Caribbean
80	*D*. *maculatus*	Estero at Playa el Arapito	ArapVE	Venezuela	Atlantic	3	0	0	Caribbean
81	*D*. *maculatus*	Tacarigua de la Laguna	TacaVE	Venezuela	Atlantic	1	1	1	Caribbean
82	*D*. *maculatus*	Chirimena	ChirVE	Venezuela	Atlantic	3	0	0	Caribbean
83	*D*. *maculatus*	Malabe, Boca del Río, Margarita	MalaVE	Venezuela	Atlantic	3	3	3	Caribbean
84	*D*. *lebretonis*	Cameroon, site AT4758	CAM	Cameroon	Atlantic	1	1	1	Cameroon
	*Eleotris senegalensis*	Cameroon, site AT4758	CAM	Cameroon	Atlantic	KU764787	KU765047	KU958383	Outgroup
85	*Gobiomorus dormitor*	Río Camoapa	CamoNI	Nicaragua	Atlantic	KU764788	KU765048	KU958382	Outgroup

### DNA sequencing

Whole genomic DNA was extracted from all of the tissue samples using a standard proteinase K and phenol-chloroform protocol [32] or Qiagen DNeasy Tissue Kits (Qiagen, Inc., Valencia, CA, USA). The mitochondrial Cytochrome *b* gene (Cyt*b*) was amplified by polymerase chain reaction (PCR) using the primers GluDG [33] and H16460 [34]. Additionally, a subset of samples representative of the genetic variation found in the Cyt*b* gene tree were sequenced using the primers RhF193 and RhR1039 [35] and the primers BactFor and BactRev [36] for the nuclear Rhodopsin (Rh) and Beta actin (β-actin) genes, respectively. PCRs were carried out in 25 μL volume reactions containing the following: 10X reaction buffer, 0.5 μM each primer, 0.2 mM dNTP, 2 mM MgCl_2_ and 1U of Taq DNA polymerase (Invitrogen). Thermocycling conditions consisted in an initial denaturation step at 94°C (2 min) followed by different time and temperature cycles depending on the gene: 35 cycles of denaturation at 94°C (45 s), annealing at 46°C (1 min) and extension at 72°C (90 s) for Cyt*b*; 5 cycles of denaturation at 94°C (30 s), annealing at 50°C (45 s) and extension at 72°C (45 s), followed by 35 cycles of denaturation at 94°C (30 s), annealing at 54°C (45 s) and extension at 72°C (45 s) for Rh; 35 cycles of denaturation at 94°C (30 s), annealing at 55°C (40 s), extension at 72°C (90 s) for β-actin and a final extension at 72°C (5 min) in all cases. All gene fragments were sequenced in both directions using the same PCR primers. Sequencing was performed by MACROGEN Inc. (Korea) sequencing service, High-Throughput Genomics Unit sequencing service (USA) and the Smithsonian Tropical Research Institute sequencing facility (Panama). Chromatograms were visually examined and then edited and assembled using Bioedit 7.2.5 [37]. DNA sequences are available in the GenBank database under the following accession numbers: KU764787-KU765046 for Cyt*b*, KU765047-KU765129 for Rh and KU958382-KU958464 for β-actin.

### Phylogenetic inference

The evolutionary substitution models that best fit our data were determined for each gene using jModeltest2 [38] and the Akaike information criterion (AIC, Table 2). Once best-fit models were determined, they were used in all of the subsequent analyses. Phylogenetic hypotheses were independently inferred for each molecular marker, nuclear data set (Rh + β-actin) and the complete concatenated data set (Cyt*b* + Rh + β-actin). Concatenated data sets only included individuals for which all genes were successfully sequenced. Maximum likelihood (ML) trees were generated using RAxMLBlackBox [39]. Genes were considered as individual partitions and treated independently with respect to evolutionary models and the optimization of branch lengths. Node support was assessed using 100 bootstrap ML replicates. Bayesian inference (BI) analyses were performed using MrBayes v.3.2.2 [40] via the CIPRES portal [41]. Each gene was considered as a distinct partition with unlinked maximum likelihood models. Two simultaneous Markov chain Monte Carlo (MCMC) searches were completed with four chains for 1 x 10^7^ generations, and trees were sampled every 1000 generations with the first 25% of the trees discarded as burn-in. Convergence between runs was assessed by monitoring the standard deviation of split frequencies with MrBayes v.3.2.2 and by using the effective sampling size (ESS) criterion in Tracer v.1.6 [42].

**Table 2:** Gene information and selected evolutionary models.

Gene	Sample size	Sequence length (bp)	Variable sites	Parsimony informative sites	Substitution model
Cytochrome b (Cyt*b*)	258	1041	306 (29.5%)	202 (19.4%)	GTR+I+G
Rhodopsin (Rh)	81	831	38 (4.6%)	19 (2.3%)	HKY
Beta actin (β-actin)	81	972	52 (5.3%)	34 (3.5%)	K80

Mean uncorrected genetic *p*-distances (D_*p*_) and their associated standard errors (S.E., 1000 bootstrap replicates) were calculated for the three genes independently and for the complete concatenated data set (Cyt*b* + Rh + β-actin) within and between clades using MEGA v.6 [43].

### Species tree and estimates of divergence time

The time to the most recent common ancestor (TMRCA) and confidence intervals (95% highest posterior density: HPD) were estimated for each clade in the Cyt*b* data set using a relaxed molecular clock with an uncorrelated lognormal distribution of rates in BEAST v.1.8.0 [44]. A Yule speciation model was assumed as a tree prior (i.e., a constant rate of speciation per lineage [45]). Two independent analyses were performed by running the MCMC for 5 x 10^7^ generations, with trees sampled every 5000 generations. The runs were examined for convergence and adequate ESS using Tracer v.1.6 and combined using LogCombiner v.1.8.0 with a burn-in fraction of 10%. The final consensus tree was produced using TreeAnnotator v.1.8.0.

To calibrate the molecular clock, we used previously published sequence and fossil record data of Gobiiformes (S1 Table). Because the Yule speciation model assumes that each tip of the tree represents one species, we selected one sequence from each of the main lineages of *Dormitator* here generated and included 37 sequences from 32 genera of Gobiiformes and two genera of Kurtiformes (S2 Table) to infer a molecular phylogeny anchored by four calibration points derived from six fossil species of Gobiiformes, with mean ages ranging from 12.5 to 52 million years ago (Mya) (S1 Table). To account for uncertainties in fossil dates or conflicts between fossils and molecules [46], fossil data points were included as soft calibration points using lognormal prior distributions in the stem nodes of interest.

Also, we estimated divergence times among *Dormitator* clades using the multispecies coalescent method *BEAST implemented in BEAST v.1.8.0. This method estimates a species tree while taking into account variation among gene trees [47]. We ran *BEAST using all sequences from all individuals and assigning them to five species, corresponding to the monophyletic clades obtained in the previous gene-tree phylogenetic hypothesis.

We used a relaxed lognormal molecular clock and a Birth-Death speciation model for each gene tree. We calibrated the molecular clock, incorporating as normal prior the substitution rate of 5.49 x 10^−2^ substitutions/site/million years (S.D. = 2.94 x 10^−2^) estimated for the Cyt*b* gene in the fossil calibrated phylogeny of Gobiiformes described above, and estimated the substitution rate of Rh and β-actin genes relative to Cyt*b*. We performed two independent MCMC runs, each for 5 x 10^7^ generations, sampling every 5000 iterations. Each run was checked for convergence and adequate ESS sampling in Tracer v.1.8, combined using LogCombiner v.1.8.0 and summarized with TreeAnnotator v.1.8.0. All BEAST v.1.8.0 analyses were run in the CIPRES portal.

## Results

### Mitochondrial and nuclear gene trees

**Fig 2:**
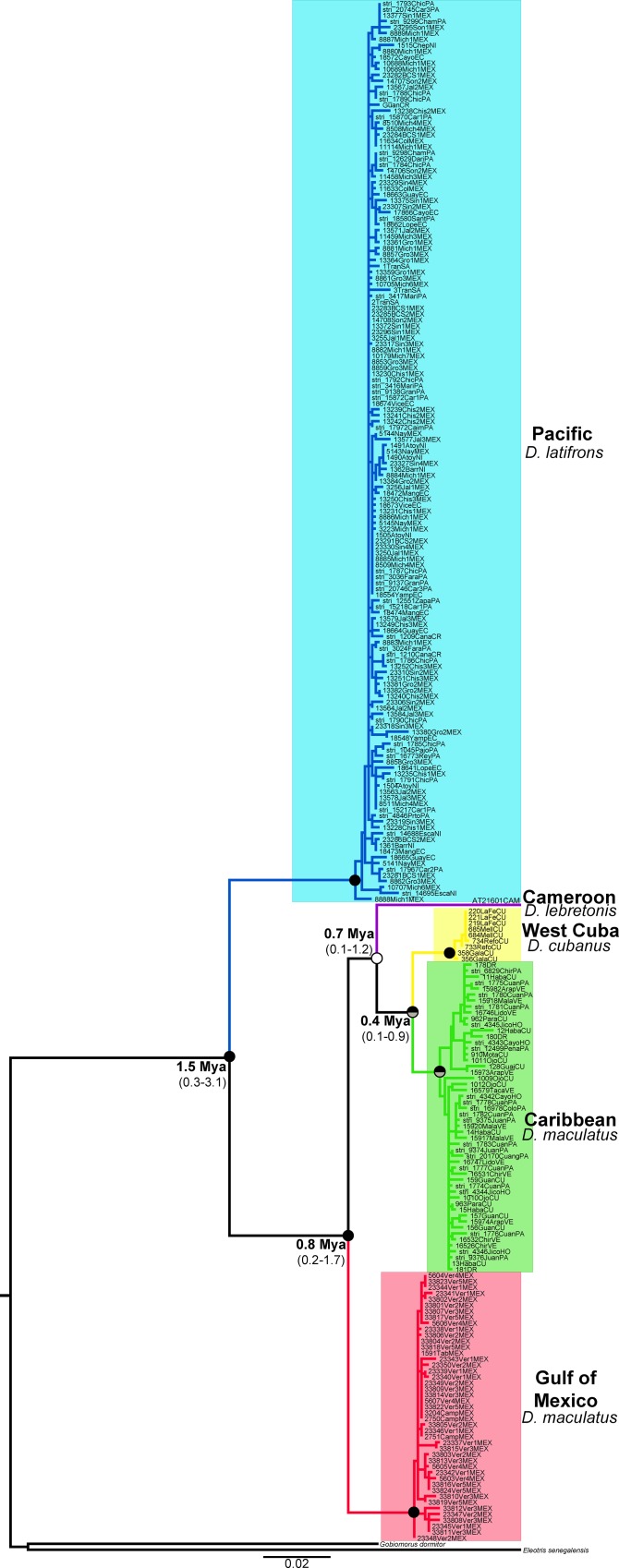
Phylogenetic hypothesis of *Dormitator* based on mitochondrial Cyt*b* gene sequences. The bullets on the nodes indicate posterior probabilities (Pp) rendered by the BI analysis (upper half) and bootstrap support (Bs) for the ML analysis (lower half). Black: high support (Pp ≥ 0.99, Bs ≥ 90%), gray: good support (Pp ≥ 0.90, Bs ≥ 65%), and white: low (Pp < 0.90, Bs < 65%). Numbers at the left of the nodes indicate the estimated mean TMRCA in Mya for each node, and between parenthesis the 95% HPD confidence intervals for each estimated date. The colors of the clades correspond to the geographic origin of the sample locations (colored dots in Fig 1).

The mitochondrial Cyt*b* gene was sequenced for 258 individuals of *Dormitator* (Table 1), which showed 306 variable positions among the 1041 base pairs (bp) sequenced (see Table 2 for additional polymorphism information and selected evolutionary models). The ML and BI Cyt*b* gene trees produced identical topologies (Fig 2) that showed two largely divergent (D_*p*_ = 8.8%, S.E. = 0.8%) and highly supported lineages (bootstrap support, Bs = 100, and Bayesian posterior probability, Pp = 100). One lineage included all specimens identified as *D*. *latifrons* from the Pacific coast, and the other lineage included all specimens from the Atlantic coast, including *D*. *maculatus*, *D*. *cubanus* and *D*. *lebretonis*. The Pacific lineage showed a complete lack of phylogeographic structure from North Mexico to Ecuador. Conversely, four geographically structured clades were recovered in the Atlantic lineage with a within-group mean sequence divergence of D_*p*_ = 3% (S.E. = 0.3%). One clade in the Gulf of Mexico was formed by *D*. *maculatus* and represented the sister group to the other three clades (D_*p*_ = 4.7–6.7%, Table 3). The West Cuba clade included individuals from Isla de la Juventud and the province of Pinar del Río on the island of Cuba, the type locality of *D*. *cubanus*. The West Cuba clade was sister to the Caribbean clade (D_*p*_ = 2.8%, S.E. = 0.5, Table 3), which included all of the remaining Western Atlantic and Caribbean *D*. *maculatus* samples from the Dominican Republic, Cuba, Honduras, Nicaragua, Panama and Venezuela. The sample of *D*. *lebretonis* from Cameroon was recovered as the sister of the West Cuba and Caribbean clades; however, the support for this finding was low.

**Table 3:** Mean *p*-distances between and within *Dormitator* clades obtained in the present phylogenetic analysis.

	Pacific lineage	Gulf of Mexico	Cameroon	Caribbean	West Cuba
**Pacific lineage**	**0.6 (0.1)**	4.1 (0.3)	4.8 (0.4)	3.9 (0.4)	4.0 (0.4)
**Gulf of Mexico**	8.9 (0.9)	**0.5 (0.1)**	2.7 (0.3)	1.8 (0.2)	2.1 (0.2)
**Cameroon**	10.3 (1.0)	6.7 (0.8)	**N/C**	2.5 (0.3)	2.7 (0.3)
**Caribbean**	8.6 (0.8)	4.7 (0.6)	6.1 (0.7)	**1.1 (0.2)**	1.3 (0.2)
**West Cuba**	8.6 (0.8)	4.8 (0.6)	5.9 (0.7)	2.8 (0.5)	**0.2 (0.1)**

Mean *p*-distances (D_*p*_) in percentages of the Cyt*b* gene (under the diagonal) and the concatenated data set Cyt*b* + Rh + β-actin (over the diagonal) between clades. The values in the diagonal (in bold) correspond to the distance within each clade for the Cyt*b* gene. N/C = Not calculated. The numbers between parentheses are standard errors.

**Fig 3:**
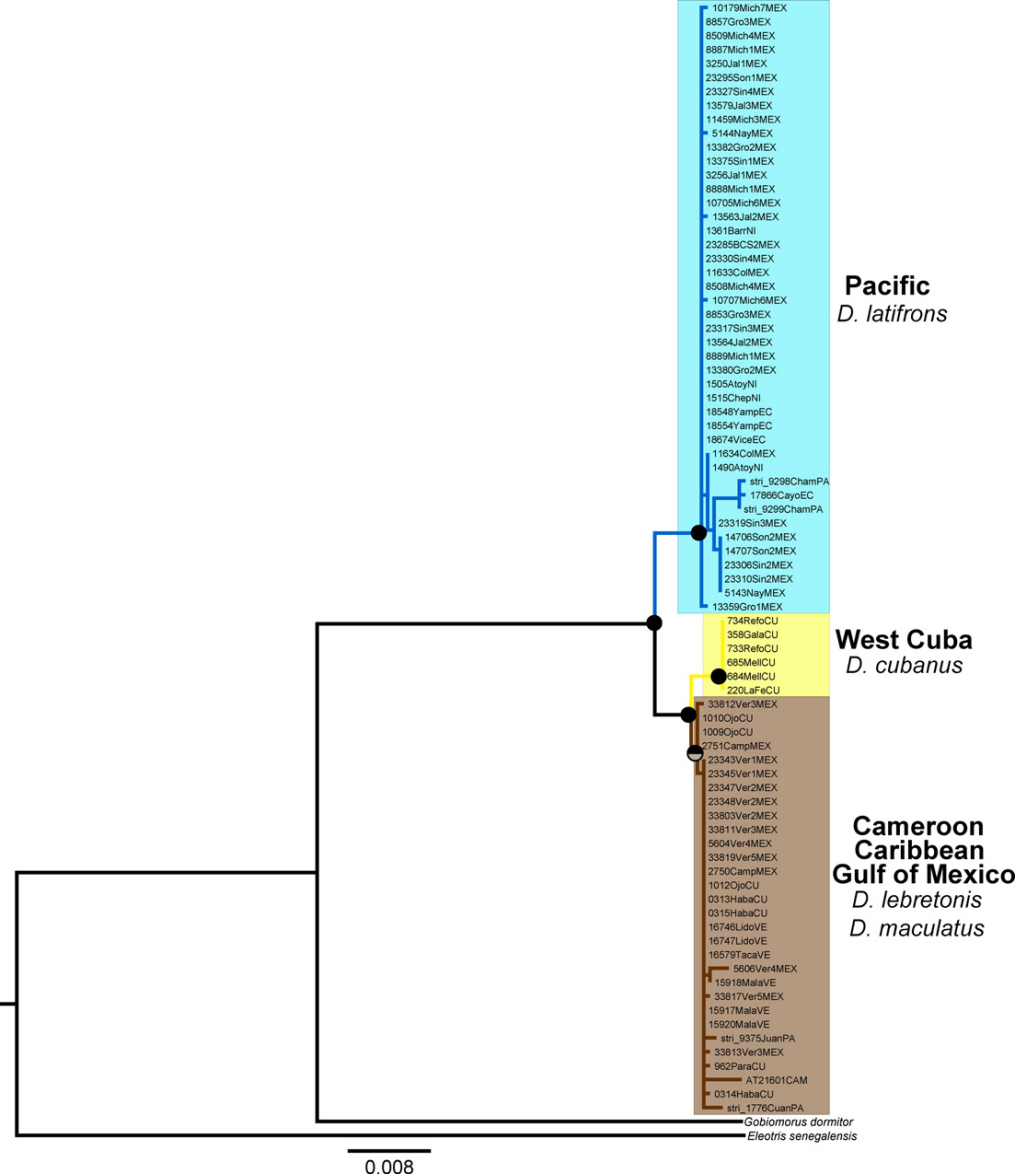
Phylogenetic hypothesis of *Dormitator* based on nuclear genes (Rh + β-actin) sequences. The bullets on the nodes indicate posterior probabilities (Pp) rendered by the BI analysis (upper half) and bootstrap support (Bs) for the ML analysis (lower half). Black: high support (Pp ≥ 0.99, Bs ≥ 90%) and gray: good support (Pp ≥ 0.90, Bs ≥ 65%). Colors of the clades: blue = Pacific, yellow = West Cuba, and brown = Gulf of Mexico + Cameroon + Caribbean.

The Rh gene (831 bp) was sequenced in 81 individuals and included 38 variable sites, whereas the β-actin gene (972 bp) was sequenced in 81 individuals and included 52 variable sites (Tables 1 and 2). The selected evolutionary models for the nuclear markers are shown in Table 2. The ML and BI reconstructions revealed the same topology for each nuclear gene separately (data not shown). Here, we present the phylogenetic hypothesis generated by the Rh + β-actin concatenated data set (Fig 3).

The nuclear (Rh + β-actin) phylogenetic hypothesis was consistent with the Cyt*b* gene tree, revealing the two Pacific and Atlantic major lineages with high support (Pp ≥ 0.99, Bs ≥ 90%) and with genetic divergences ranging from D_*p*_ = 0.8% (S.E. = 0.3%) to D_*p*_ = 1.6% (S.E. = 0.4%) for Rh and β-actin, respectively. However, within the Atlantic lineage, only two clades were recovered by both nuclear genes: the West Cuba clade and the remaining Atlantic *Dormitator* samples, including *D*. *maculatus* from the Gulf of Mexico and the Caribbean and *D*. *lebretonis* from Cameroon. Average D_*p*_ values between these Atlantic clades ranged from 0.5% (S.E. = 0.2%) to 0.4% (S.E. = 0.2%) for Rh and β-actin, respectively.

**Fig 4:**
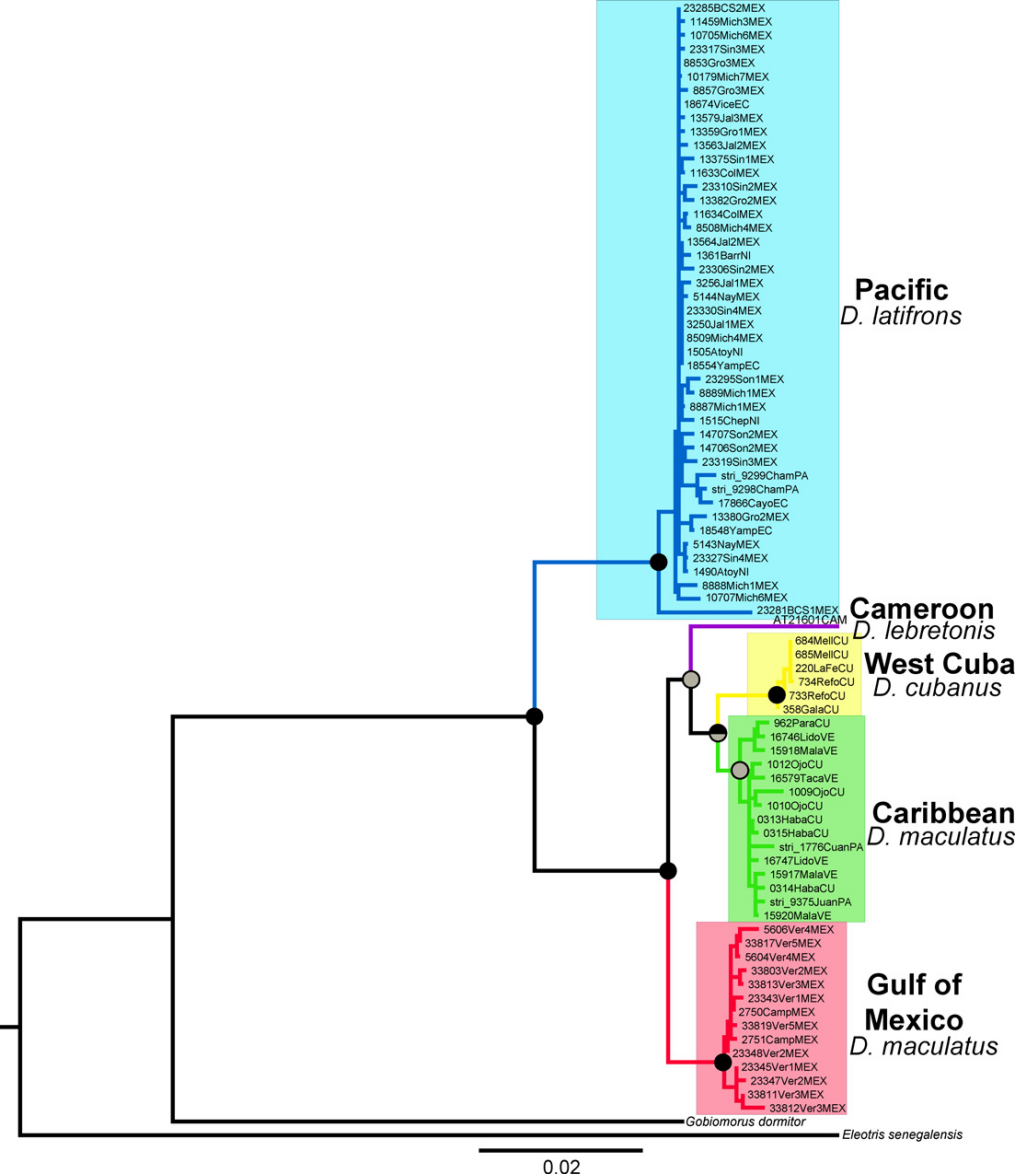
Phylogenetic hypothesis of *Dormitator* based on the concatenated mitochondrial and nuclear (Cyt*b* + Rh + β-actin) genes sequences. The bullets on the nodes indicate posterior probabilities (Pp) rendered by the BI analysis (upper half) and bootstrap support (Bs) for the ML analysis (lower half). Black: high support (Pp ≥ 0.99, Bs ≥ 90%) and gray: good support (Pp ≥ 0.90, Bs ≥ 65%). The colors of the clades correspond to the geographic origin of the sample locations (colored dots in Fig 1).

The complete concatenated data set (Cyt*b* + Rh + β-actin) analysis (Fig 4) produced a topology that was congruent with that of the previously described Cyt*b* gene tree (Fig 2), with the highest support for the African *D*. *lebretonis* sister relationship occurring with the West Cuba and Caribbean clades. The Pacific and Atlantic lineages showed an overall D_*p*_ of 4.0% (S.E. = 0.4%).

### Species tree and molecular dating

The molecular clock analysis results indicated a mean TMRCA between the Pacific and Atlantic lineages of 1.5 Mya (95% HPD: 0.3–3.1 Mya), which corresponded to the Mid- to Late Pliocene period (1.75–5.3 Mya) (Fig 2, S1 Fig). The divergence times among the Atlantic lineage clades were all recent and showed overlapping confidence intervals, indicating that all of these cladogenetic events occurred during the Pleistocene (95% HPD ranging between 0.1–1.7 Mya). The TMRCA ranged from 0.8 Mya (95% HPD: 0.2–1.7 Mya) for the split between the Gulf of Mexico and remaining Atlantic clades (Cameroon, West Cuba and Caribbean) to 0.4 Mya (95% HPD: 0.1–0.9 Mya) for the West Cuba and Caribbean clades. The mean divergence of the Africa and Western Atlantic clades was estimated at 0.7 Mya (95% HPD: 0.1–1.2 Mya) (Fig 2, S1 Fig).

**Fig 5:**
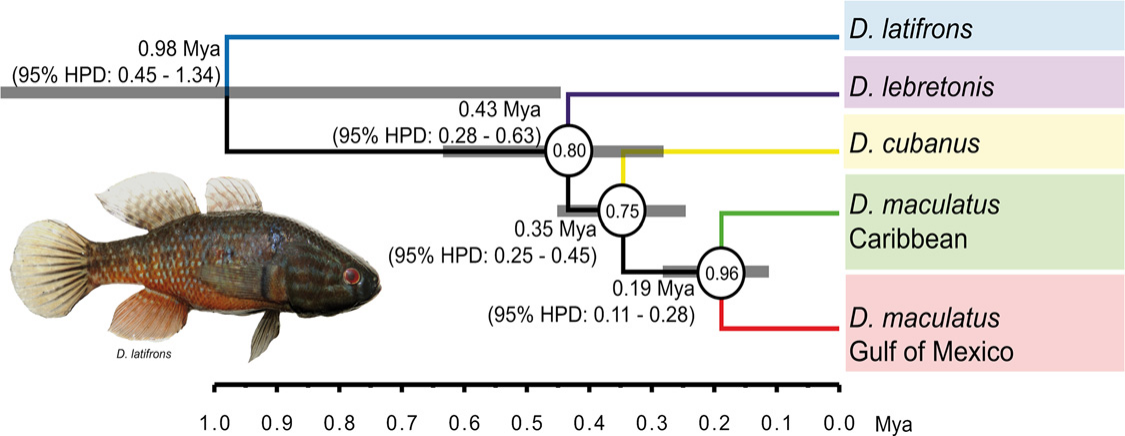
Time-calibrated *Dormitator* species tree hypothesis, based on the multispecies coalescent analyses of Cyt*b*, Rh and β-actin genes. Numbers at the left of the nodes indicate the estimated mean TMRCA in Mya for each node, and horizontal grey bars at nodes represent the 95% HPD intervals for each estimated date. Node circles indicate posterior probabilities values (Pp) for the *BEAST analysis. The scale bar below the tree shows time in Mya. The colors of the clades correspond to the geographic origin of the sample locations (colored dots in Fig 1). Image of *D*. *latifrons* was obtained from the CPUM-Universidad Michoacana de San Nicolás de Hidalgo, México; voucher specimen No. 24632.

The species-tree hypothesis agreed with the mitochondrial and concatenated gene trees in recovering the Pacific and Atlantic *Dormitator* as reciprocally monophyletic groups but differed regarding the relationships among Atlantic *Dormitator* taxa (Fig 5). *Dormitator lebretonis* from Cameroon was recovered in a basal position from the remaining Atlantic species (Pp = 0.75). Also, *D*. *maculatus* from the Gulf of Mexico and the Caribbean were recovered as sister taxa (Pp = 0.96), and *D*. *cubanus* was sister to them (Pp = 0.75). Divergence time estimates for these cladogenetic events were younger, although similar and with overlapping 95% HPD, than the estimates obtained for the fossil calibrated Cyt*b* gene tree (Fig 2, S1 Fig). The main event that split the Atlantic and Pacific lineages was dated ~0.98 Mya (95% HPD: 0.45–1.34), and the divergence between *D*. *lebretonis* and the western Atlantic taxa was dated at 0.43 Mya (95% HPD: 0.28–0.63). Within the western Atlantic, mean divergence times ranged between 0.19 and 0.35 Mya.

## Discussion

Species show different responses to common geological and environmental processes. As a consequence, their resulting evolutionary patterns may also differ because they depend on the relative roles of geological and ecological factors and their interaction with species-specific life-history traits [48,49]. These differences have been widely explored and compared across a broad range of taxa in Central America (see Bacon et al. [16] for a recent review), including fish (e.g. [50–54]), and reconciled multiple pulses of dispersal and vicariance. The timing of these events was most likely associated not only with the variable availability of land and marine and freshwater corridors but also with the establishment of suitable climates and environments during the formation of the Isthmus [16]. However, how these factors affect the evolutionary patterns of amphidromous fish like *Dormitator* species had never been addressed before.

Species with an amphidromous life-history strategy would be expected to show different evolutionary patterns compared to strictly marine or freshwater species [10,55,56]. Contrary to this prediction, and despite the supposed capability of its larvae for long-distance dispersal through marine environments, *Dormitator* showed many evolutionary similarities with other freshwater fish, such as an absence of phylogeographic structure on the Pacific coast and a high phylogeographic structure in the Atlantic. Furthermore, the estimated time of vicariance between ocean basins was among the most recent reported to date, suggesting that gene flow existed between oceans until the very last stages of formation of the Central American Isthmus in the late Pliocene-Pleistocene period.

### Pacific-Atlantic split

The closure of the Central American Isthmus is the major vicariant event that shaped the phylogenetic structure of *Dormitator*. The finding of a Pacific-Atlantic divergence rather than the expected Africa-America divergence rejects the hypothesis that the evolutionary patterns of *Dormitator* can be explained only by vicariance following the break-up of Gondwana. In contrast, this pattern suggests a widespread ancestral distribution of the genus or an American origin with dispersal to Africa following the closure of the Isthmus. Early transisthmian differentiation led to the formation of two main lineages of *Dormitator*: one lineage composed of the species from the Pacific basin (*D*. *latifrons*) and the other lineage composed of the species sampled in the Atlantic basin (*D*. *maculatus*, *D*. *cubanus* and *D*. *lebretonis*). This relationship was independently recovered by both mitochondrial and nuclear genes, even though the latter have been considered to evolve at a rate not fast enough to resolve transisthmian divergences [15]. Here, despite their slower substitution rate and higher effective population size, nuclear loci provided a clear pattern of divergence that indicated a complete interruption of gene flow between oceans.

The divergence between the Pacific and Atlantic Cyt*b* lineages of *Dormitator* was estimated at between 0.3 and 3.1 Mya, which is consistent with a final closure of the Central American Seaway during the Late Pliocene (*ca*. 2.8 Mya, sensu Coates et al. [57]). The species tree analysis, on the other hand, estimated a younger date for this event (0.45-1-34 Mya). This pattern is expected according to the multispecies coalescent, which predicts that gene divergence will predate species divergence [58] and furthermore suggests that gene flow could have occurred across the Isthmus of Panama until very recent times during the Pleistocene. Divergence times between geminate species pairs, however, remain a subject of debate. According to Lessios [15], 30% of 115 species pairs of geminate clades (including echinoids, crustaceans, mollusks and fish) were likely to have diverged *ca*. 2.8 Mya, approximately 63% were separated at some point earlier during the long period of geological upheavals associated with the rising Isthmus, and 7% may have maintained genetic connections after the Isthmus closure. More recently, Bacon et al. [16] provided evidence that transisthmian divergence was a complex process that proceeded over at least two stages at approximately 24 Mya and 8 Mya. Furthermore, a significant decrease in the migration rates of marine organisms was detected at 2 Mya (1.03–4.35 Mya) [16], which is highly consistent with the divergence time estimated for *Dormitator*. Apparently, the large variation in divergence times is not predicted by intrinsic species biological factors. Rather, it seems that these differences are better explained by extrinsic features controlling habitat formation and availability during the geological development of the region [16]. This differentiation has been tested in the gastropod genera *Cerithium* and *Cerithidea*, in which geminate species inhabiting high intertidal mangrove habitats exhibit less evolutionary divergence than those that inhabit lower intertidal and subtidal habitats [17]. It is assumed that mangrove or estuarine habitats were the last to disappear during the Central American Seaway closure, thus allowing species that inhabited these environments, such as *Dormitator*, to maintain gene flow until the final closure of the seaway. Therefore, the divergence times of these species pairs most accurately correspond to the final completion of the Isthmus of Panama because they represent the milestone for the last connectivity events between oceans.

### Pacific lineage

*Dormitator* in the Pacific showed a total absence of phylogeographic structure from Northern Mexico to Ecuador, indicating high gene flow across the distribution range of *D*. *latifrons*. Therefore, molecular data do not support the morphological differentiation of the subspecies of *D*. *latifrons mexicanus* in the eastern Pacific [59]. The genetic homogeneity across the Pacific distribution of *Dormitator* could be a product of their amphidromous life-history strategy, which potentially allows for long-distance dispersal during the marine larval stages, and their tolerance to salinity, which could also facilitate marine dispersal during adult stages [60,61]. Reduced genetic structure across the lower Central American Pacific coast has also been reported among primary freshwater fish species, which, contrary to amphidromous fish, do not tolerate salinity [51,54,62]. In such cases, it has been proposed that the lower sea levels during the last glacial maxima may have favored population dispersal and expansion events via river anastomosis [62]. If accepted, this hypothesis would indicate that the common pattern of low phylogeographical structure across the Pacific coast is not explained by biotic factors, such as amphidromy, but rather by abiotic factors, such as geology and climatic changes.

### Atlantic lineage differentiation

The Atlantic lineage of *Dormitator* contained three species: *D*. *maculatus*, *D*. *cubanus* and *D*. *lebretonis*, all of which were reciprocally monophyletic, although their relationships showed conflicting results between the gene trees and the species tree analyses. For example, in the Cyt*b* and concatenated gene trees, *D*. *maculatus* from the Gulf of Mexico and the Caribbean represented two non-sister clades, while the species tree recovered these clades as sisters with high support. Additionally, the large genetic divergence between the two clades of *D*. *maculatus* (Cyt*b* D_*p*_ = 4.7 ± 0.6) might warrant a taxonomic revision because they might constitute cryptic species. Although cryptic evolutionary lineages are expected in widely distributed species, including amphidromous species [12], further analyses that incorporate missing species of the genus, such as *D*. *lophocephalus* from Suriname, could help to determine the validity of these species as well as their distribution ranges. The four Atlantic clades showed distinct and geographically delimited distributions, suggesting that phylogenetic structure may be the result of long-term vicariance and barriers to dispersal among basins within the Caribbean. This pattern is expected and consistent with the higher historical isolation of the Caribbean compared to the Pacific drainages [50,54,62].

#### Gulf of Mexico clade

The youngest cladogenetic event within the *Dormitator* Atlantic lineage, as inferred by the multispecies coalescence analysis, corresponds to the divergence of the Gulf of Mexico and Caribbean clades approximately 0.19 Mya (95% HPD: 0.11–0.28 Mya). This divergence could have resulted from a combination of factors and a series of Pleistocene oceanographic changes that followed the closure of the Panama Isthmus. Oceanographic changes included variations of the sea level and changes in salinity, temperature and current patterns, such as the intensification of the Loop Current, the Florida Current, the Equatorial Undercurrent and the Gulf Stream [63]. All of these factors had major effects on marine paleobiogeography and paleoceanography [64] and may have also caused the isolation of populations in the Gulf of Mexico. In particular, the Loop Current has been proposed to play an important role as a barrier between the Gulf of Mexico and the Caribbean [10]. This current moves north between the Yucatán Peninsula and Cuba and recirculates to travel between Florida and Cuba [65]. Additionally, because currents along the coast of the Gulf of Mexico are not very strong, it is unlikely that *Dormitator* could be dispersed over long distances during their marine larval stage. In addition, the Yucatán Peninsula has been largely recognized as an area with a meager fish fauna because of its lack of surface river systems [22]. Consequently, this peninsula could have constituted a barrier to dispersal between the Caribbean and the Gulf of Mexico basins [10].

The large divergence observed in *Dormitator* for the Gulf of Mexico clade is also consistent with its delimitation as a separate biogeographic region from the Greater Caribbean, which is based on shore-fishes distribution data [66] and the genetic discontinuity observed in multiple co-distributed marine taxa [67–70]. Recent climatic events are hypothesized to have strongly influenced the distributions and genetic signatures of taxa between the Atlantic and Gulf of Mexico ocean basins [68,71]. For example, the reduced sea levels associated with the glacial maxima might have led to the isolation of the western part of the Gulf of Mexico, which represents a refuge area for populations of estuarine species [67]. However, this expected phylogenetic separation between the Gulf of Mexico and the Atlantic Ocean basins is not observed in several near and offshore marine species and may not be, due to these species’ greater dispersal capabilities [67] (also see Soltis et al. [70]). Thus, it appears that vicariant scenarios alone cannot explain this phylogenetic break and the current phylogeographic pattern is likely the result of the interplay among the shared paleoclimatic history, ecological factors and species-specific life-history traits [67,68,70].

#### Cameroon

The divergence of *D*. *lebretonis* from the western Atlantic species occurred between 0.28 and 0.63 Mya. The short elapsed time since the split, as well as the retained ancestral polymorphisms or recent trans-Atlantic gene flow, could explain why this divergence was only recovered by the mitochondrial and combined data sets and not by any combination of the nuclear markers analyzed [12,15]. Similarly, differences in effective population size and gene flow rates between populations on both sides of the Atlantic might explain the discordant results of the gene and species trees [58]. Unfortunately, these hypotheses remain untested because only one sample of *D*. *lebretonis* could be included in the study. Therefore, these results must be interpreted cautiously.

The sister relationship of the African *D*. *lebretonis* and the rest of the American Atlantic clades both rejects the vicariant hypothesis of a Gondwanan origin of *Dormitator* and postdates by almost 20 million years Rosen’s [72] Eastern Atlantic/Western Atlantic generalized track hypothesis, proposing that sister species distributed across the Atlantic regions were the result of Cenozoic (65–20 million years ago) spreading across the Atlantic basin. Several groups of fish share this amphi-Atlantic distribution [73–76], and despite genetic differentiation between Atlantic coasts, certain authors consider the mid-Atlantic Barrier (> 3500 km of deep ocean) to act as a soft barrier for marine species [76]. In fact, patterns showing a greater resemblance between taxa on both sides of the Atlantic than between the western Atlantic and eastern Pacific are not uncommon for estuarine taxa. For example, for the mangroves *Avicennia germinans* and *Rhizophora mangle*, which also show current trans-Atlantic gene flow [77], a strong role of contemporary currents has been proposed in shaping their genetic landscape. The main currents are the North Equatorial Current and South Equatorial Current, which travel from east to west, and the North Equatorial Counter Current, which travels from west to east. All of them were established following the breakup of West Gondwana and have maintained the same direction connecting the Americas with West Africa since the final closure of the Central American Isthmus [78–81]. Because of the ecological and biogeographical resemblances among *Rhizophora*, *Avicennia* and *Dormitator*, it is likely that these species, as well as other amphidromous or estuarine fish (e.g., *Eleotris*), are affected by similar processes that allow long-distance dispersal through marine current drift. However, the degree of isolation and contemporary gene flow across the Atlantic should be addressed by examining a wider sample of African populations.

#### Caribbean and West Cuba clades

The island of Cuba harbored two clades of *Dormitator*, with non-overlapping distributions. The West Cuba clade was restricted to Juventud Island and Pinar del Río Province locations in the western region of Cuba, which fits with the distribution of *D*. *cubanus* according to Ginsburg [59]. The Caribbean clade included the eastern region of Cuba as well as Dominican Republic, Honduras, Nicaragua, Panama and Venezuela, and corresponded to the distribution range of *D*. *maculatus*.

The gene trees showed a closer relationship between the West Cuba clade and the Caribbean clade, while the species tree showed West Cuba as the sister taxon of *D*. *maculatus* from the Caribbean and the Gulf of Mexico clades. Additionally, the West Cuba clade was the only clade recovered by the nuclear gene trees as distinct within the Atlantic basin. This discrepancy between molecular markers and methods might suggest a selective or demographic process through which nuclear alleles evolved or were sorted faster than mitochondrial alleles. One possibility is that this process could have been favored by drift in a population with a small effective size after the isolation of western Cuba as a result of climatic changes, current shifts and sea level variations [82].

Considering the large and homogeneous distributions of most species or clades of *Dormitator*, the small geographic scale of differentiation and the highly restricted distribution of *D*. *cubanus* is remarkable. Yet the pattern and timing of cladogenesis in Cuban *Dormitator* match previous biogeographic studies on fish and amphibians that support the paleogeographic hypothesis that Juventud Island has been isolated from Cuba at least since the Oligocene and more recently during the Pleistocene period [72,83–86]. Within the endemic Cuban poeciliid tribe Girardinini, the isolation of Juventud Island and western Cuba (Guanahacabibes Peninsula at Pinar del Río Province) has been proposed as a vicariant event promoting speciation in this group of fish [85]. For example, the presence of *Quintana*, the sister group of all Girardinini, in Juventud Island could explain an ancient isolation event between the two islands. Additionally, a more recent isolation during the late Pliocene-Pleistocene would be supported by the well-differentiated populations of *Glaridichthys falcatus* in Juventud Island and central Cuba, and by *Girardinus rivasi*, which is endemic to Juventud Island [85]. These two species and populations of Juventud Island present Cyt*b* divergences from their closest relatives in Cuba (D_*p*_ = 1.7 to 2.4% between *G*. *falcatus* populations and D_*p*_ = 1.1 to 1.8% between *G*. *rivasi* and *G*. *microdactylus*) that were similar to those observed between *D*. *cubanus* and *D*. *maculatus* (D_*p*_ = 2.8±0.4%), suggesting coeval cladogenetic events. A concordant pattern was observed in the *Peltophryne* toad radiation, with reciprocally monophyletic populations in Juventud Island and Cuba (*Peltophryne empusa* and *Peltophryne peltocephala*) and species with distributions limited to western Cuba (*Peltophryne fustiger*). In this case, genetic divergences were shallow but significant (16S D_*p*_ = 0.8–0.4%, COI D_*p*_ = 2.1–0.6%) and suggest a recent Pleistocene-Holocene separation between the islands of Juventud and Cuba [86]. A similar speciation pattern might also exist in marine taxa from Juventud Island. For example, the coral reef fish *Gramma dejongi* was described as an endemic species from this region, but contrary to *Dormitator*, it shows no mitochondrial genetic differentiation relative to co-occurring congeneric species. Therefore, this difference has been proposed as a case of incipient speciation or even a local color variant of the sympatric and widespread *Gramma loreto* [87].

The deep phylogenetic differentiation observed in the Atlantic lineage supports the current species of *Dormitator* and provides evidence for possible cryptic species. Even the lowest sequence divergence among Atlantic clades, which was observed between the West Cuba and Caribbean clades (Cyt*b* D_*p*_ = 2.8%), largely exceeds the 2% sequence divergence generally accepted as a cut-off value between sister species of vertebrates [88]. Our molecular data support the validity of *D*. *cubanus*, the distribution of which is consistent with the original description of the species [59] from Pinar del Río and extends its range to Juventud Island. The parapatric occurrence of *D*. *cubanus* with *D*. *maculatus* in Cuba was also noted, although more recent works had exclusively indicated the presence of *D*. *maculatus* in all the Cuban territory [89,90].

## Conclusion

This work is the first study to address the evolutionary history of the amphidromous fish genus *Dormitator*. Despite the expected long-distance dispersal capability of these species based on their amphidromous life-style, vicariant geological processes rather than life-history traits appear to be the main drivers of speciation in *Dormitator*. First, the presence of geminate lineages of *Dormitator* across the Pacific and Atlantic slopes confirms the importance of the Central American Isthmus in the evolutionary history of the genus. Second, the four geographically delimited Atlantic clades appear to be shaped by isolation processes that are largely congruent with geologic and oceanographic events related to the closure of the Central America Isthmus. The high genetic structure and isolation of the Caribbean basin is consistent with patterns observed in both primary freshwater and marine shore fish and further contrasts with the genetic homogeneity along the Pacific coast. This pattern is shared among other species with much lower dispersal capabilities and is likely caused by enhanced river connectivity during periods with lower sea levels in the last glacial maximum. Comparative studies of other fish sympatric to *Dormitator* could provide further information on the relative roles of abiotic (i.e., geological and climatic) and biotic processes (i.e., species ecology and life-history traits) in the evolutionary history and biogeographic patterns of amphidromous neotropical fishes.

## Supporting Information

S1 Fig Time-calibrated phylogenetic reconstruction of *Dormitator* and 37 Gobiiformes taxa based on mitochondrial Cyt*b* sequences. Within *Dormitator* (colored area), horizontal grey bars represent the 95% highest posterior density of the estimated time to the most recent common ancestor (TMRCA) in Mya for each node. Numbers at the left of the grey bars indicate the estimated mean TMRCA. The colors of the clades correspond to the geographic origin of the sample locations (as in Fig 1). Bullets represent posterior probability support for each node. Black bullets represent Pp = 1, and white bullets represent Pp = 0.6. Red circles represent the four fossil calibrations used to time-constrain each node in the molecular clock analysis (S2 Table). The scale bar below the tree shows time in Mya.
(EPS)

S1 Table Fossil species of Gobiiformes used to calibrate the *Dormitator* molecular clock analysis. (DOCX)

S2 Table Species, GenBank accession number and fossil calibration points for each individual included in the *Dormitator* molecular clock analysis based on cytochrome *b* (Cyt*b*) sequences. (DOCX)
