# Supplementary material for: Molecular Phylogeny and Biogeography of the Amphidromous Fish Genus Dormitator Gill 1861 (Teleostei: Eleotridae)
Source: PLoS One. 2016 Apr 13;11(4):e0153538. doi: 10.1371/journal.pone.0153538 (PMC4830628; doi:10.1371/journal.pone.0153538)
Supplement: S1 Table — (DOCX) [file pone.0153538.s002.docx]

**S1 Table. Fossil species of Gobiiformes used to calibrate the *Dormitator* molecular clock analysis.**

| **Calibration** | **Fossil** | **Formation or geographic locale(s)** | **Formation age** | **Age (Mya)** | **Reference** |
| --- | --- | --- | --- | --- | --- |
| C1 | *Lepidocottus papyraceus* (Agassiz) | Italy | Lower Oligocene | 35 | [1] |
| C1 | *Lepidocottus gracilis* Laube | Eastern Germany | Lower Oligocene | 35 | [1] |
| C2 | *Gobiidarum nolfi* n.sp. | Gujarat, India | Lower Eocene | 52 | [2] |
| C2 | *Gobiidarum vastani* n.sp. | Gujarat, India | Lower Eocene | 52 | [2] |
| C3 | *Gobiomorphus* | Central Otago, New Zealand | Early Miocene | 20-16 | [3] |
| C4 | *Gobius multipinnatus* Meyer, 1848 | Italy | Mid Miocene | 15-10 | [4] |

**S1 Table references**

1. Gierl C, Reichenbacher B, Gaudant J, Erpenbeck D, Pharisat A. An extraordinary gobioid fish fossil from Southern France. PLOS ONE. 2013;8: e64117. doi: 10.1371/journal.pone.0064117.

2. Bajpai S, Kapur VV. Oldest known gobiids from vastan lignite mine (early Eocene), Surat district, Gujarat. Curr Sci. 2004;87: 433–435.

3. McDowall RM, Kennedy EM, Lindqvist JK, Lee DE, Alloway BV, Gregory MR. Probable *Gobiomorphus* fossils from the Miocene and Pleistocene of New Zealand (Teleostei: Eleotridae). J R Soc N Z. 2006;36: 97-99. doi: 10.1080/03014223.2006.9517803.

4. Simonović PD. Phylogenetic relationships of the Ponto-Caspian gobies and their relationships to the Atlantic-Mediterranean Gobiinae. J Fish Biol. 1999;54: 533-555.
