# Supplementary material for: Molecular Phylogeny and Biogeography of the Amphidromous Fish Genus Dormitator Gill 1861 (Teleostei: Eleotridae)
Source: PLoS One. 2016 Apr 13;11(4):e0153538. doi: 10.1371/journal.pone.0153538 (PMC4830628; doi:10.1371/journal.pone.0153538)
Supplement: S2 Table — (DOCX) [file pone.0153538.s003.docx]

**S2 Table. Species, GenBank accession number and fossil calibration points for each individual included in the *Dormitator* molecular clock analysis based on cytochrome *b* (Cyt*b*) sequences.**

| **Species** | **Calibration point** | **Cyt*b*** | **Reference** |
| --- | --- | --- | --- |
| *Acentrogobius chlorostigmatoides* | C2 | NC020346 | [1] |
| *Acentrogobius pflaumii* | C2 | NC018064 | [2] |
| *Amoya chusanensis* | C2 | NC020347 | [3] |
| *Awaous guamensis* | C2 | JN387630 | [4] |
| *Boleophthalmus boddarti* | C2 | NC023468 | [5] |
| *Bostrychus sinensis* | C1, C2 | AY722236 | [6] |
| *Butis butis* | C1, C2 | KF415524 | [7] |
| *Chaeturichthys stigmatias* | C2 | NC020786 | [8] |
| *Chromogobius zebratus* | C2, C4 | FJ526783 | [9] |
| *Eleotris acanthopoma* | C2 | AP004455 | [10] |
| *Gillichthys mirabilis* | C2 | NC012906 | Bentley and Wiley (unpublished) |
| *Glossogobius circumspectus* | C2 | NC018824 | Xu, Jin, and Wang (unpublished) |
| *Gobiomorphus coxii* | C2, C3 | AY722220 | [6] |
| *Gobiomorphus hubbsi* | C2, C3 | AY722227 | [6] |
| *Gobius niger* | C2 | FJ526782 | [9] |
| *Kraemeria cunicularia* | C2 | AB021250 | [11] |
| *Kribia nana* | C1, C2 | AY722222 | [9] |
| *Lophiogobius ocellicauda* | C2 | NC020783 | [12] |
| *Luciogobius platycephalus* | C2 | NC019811 | [13] |
| *Millerigobius microcephalus* | C2, C4 | KF415611 | [7] |
| *Odontamblyopus rubicundus* | C2 | NC019647 | [14] |
| *Odontobutis potamophila* | C2 | KF874495 | Li, Liu, Li, and Kuang (unpublished) |
| *Ophiocara porocephala* | C1, C2 | AY722250 | [6] |
| *Oxyeleotris marmorata* | C1, C2 | AY722252 | [6] |
| *Oxyeleotris selheimi* | C1, C2 | AY722238 | [6] |
| *Oxyurichthys formosanus* | C2 | NC020345 | [15] |
| *Pseudogobius javanicus* | C2 | NC022186 | [16] |
| *Rhinogobius giurinus* | C2 | NC022692 | [17] |
| *Rhyacichthys aspro* | C2 | AP004454 | [10] |
| *Scartelaos histophorus* | C2 | NC017888 | [18] |
| *Sicyopterus japonicas* | C2 | NC018826 | [19] |
| *Sicyopterus lagocephalus* | C2 | NC022838 | [20] |
| *Stiphodon alcedo* | C2 | NC018054 | [21] |
| *Synechogobius ommaturus* | C2 | NC020767 | [22] |
| *Tridentiger barbatus* | C2 | NC018823 | [14] |
| *Kurtus gulliveri* |  | EU380974 | [23] |
| *Apogon semilineatus* |  | NC022510 | [24] |

**S2 Table references**

1. Jin X, Liu X, Sun Y. Complete mitochondrial genome of the Greenspot goby *Acentrogobius chlorostigmatoides* (Perciformes, Gobioidei): repetitive sequences in the control region. Mitochondrial DNA. 2013;24: 400-402. doi: 10.3109/19401736.2013.763244.
2. Jin X, Wang R, Zhao S, Xu T, Shi G. Complete mitochondrial genome of the striped sandgoby *Acentrogobius pflaumii* (Perciformes, Gobiidae). Mitochondrial DNA. 2012;23: 420-422. doi: 10.3109/19401736.2012.710213

3. Jin X, Zhao S, Sun Y. Complete mitochondrial genome sequence and structure of control region of the *Amoya chusanensis* (Perciformes, Gobioidei). Mitochondrial DNA. 2013;24: 403-405. doi: 10.3109/19401736.2013.763245.

4. Lindstrom DP, Blum MJ, Walter RP, Gagne RB, Gilliam JF. Molecular and morphological evidence of distinct evolutionary lineages of *Awaous guamensis* in Hawaii and Guam. Copeia. 2012;2012: 293-300.

5. Zhang YT, Ghaffar MA, Li Z, Chen W, Chen SX, Hong WS. Complete mitochondrial genome of the mudskipper *Boleophthalmus boddarti* (Perciformes, Gobiidae). Mitochondrial. DNA. 2014 Jan 17. doi: 10.3109/19401736.2013.873901.

6. Thacker CE, Hardman MA. Molecular phylogeny of basal gobioid fishes: Rhyacichthyidae, Odontobutidae, Xenisthmidae, Eleotridae (Teleostei: Perciformes: Gobioidei). Mol Phylogenet Evol. 2005;37: 858-871. doi: 10.1016/j.ympev.2005.05.004.

7. Agorreta A, San Mauro D, Schliewen U, Van Tassell JL, Kovačić M, Zardoya R, et al. Molecular phylogenetics of Gobioidei and phylogenetic placement of European gobies. Mol Phylogenet Evol. 2013;69: 619-633. doi: 10.1016/j.ympev.2013.07.017.

8. Sun Y, Jin X, Zhao S, Liu X. Mitochondrial genome of *Cryptocentrus yatsui* (Perciformes, Gobioidei) and phylogenetic consideration within the genus Cryptocentrus. Mitochondrial DNA. 2013;24: 376-378. doi: 10.3109/19401736.2013.763236.

9. Neilson ME, Stepien CA. Escape from the Ponto-Caspian: evolution and biogeography of an endemic goby species flock (Benthophilinae: Gobiidae: Teleostei). Mol Phylogenet Evol. 2009;52: 84-102. doi: 10.1016/j.ympev.2008.12.023.

10. Miya M, Takeshima H, Endo H, Ishiguro NB, Inoue JG, Mukai T, et al. Major patterns of higher teleostean phylogenies: a new perspective based on 100 complete mitochondrial DNA sequences. Mol Phylogenet Evol. 2003;26: 121-138. doi: 10.1016/S1055-7903(02)00332-9.

11. Akihito IA, Iwata A, Kobayashi T, Ikeo K, Imanishi T, Ono H, et al. Evolutionary aspects of gobioid fishes based upon a phylogenetic analysis of mitochondrial cytochrome b genes. Gene. 2000;259: 5-15. doi: 10.1016/S0378-1119(00)00488-1.

12. Quan X, Jin X, Sun Y. The complete mitochondrial genome of *Lophiogobius ocellicauda* (Perciformes, Gobiidae). Mitochondrial DNA. 2014;25: 95-97. doi: 10.3109/19401736.2013.784753.

13. Jin X, Sun Y, Zhao S, Wang R. Mitochondrial genome of the *Luciogobius platycephalus* (Perciformes, Gobioidei). Mitochondrial DNA. 2013;24: 379-381. doi: 10.3109/19401736.2013.763237.

14. Liu T, Jin X, Wang R, Xu T. Complete sequence of the mitochondrial genome of *Odontamblyopus rubicundus* (Perciformes: Gobiidae): genome characterization and phylogenetic analysis. J Genet. 2013;92: 423-432. doi: 10.1007/s12041-013-0283-6.

15. Sun Y, Wei T, Jin X. Unusual features of control region and a novel NADH 6 genes in mitochondrial genome of the finespot goby, *Chaeturichthys stigmatias* (Perciformes, Gobiidae). Mitochondrial. DNA. 2013 Oct 9. doi: 10.3109/19401736.2013.840598.

16. Huang SP, Shen CN, Chen IS. The complete mitochondrial genome of the Java fat-nose goby *Pseudogobius javanicus* (Teleostei, Gobiidae). Mitochondrial. DNA. 2013;26: 159-161. doi: 10.3109/19401736.2013.819502.

17. Xie L, Yang X, Ma Z, Yang RC. Complete mitochondrial genome of *Rhinogobius giurinus* (Perciformes: Gopbiidae: Gobionellinae). Mitochondrial. DNA. 2015;26: 321-322. doi: 10.3109/19401736.2013.830293.

18. Quan X, Jin X, Wang R, Xu T, Shi G. Complete mitochondrial genome of the walking goby *Scartelaos histophorus* (Perceformes, Gobiidae). Mitochondrial DNA. 2012;23: 298-300. doi: 10.3109/19401736.2012.674127.

19. Chiang TY, Chen IS, Lin HD, Chang WB, Ju YM. Complete mitochondrial genome of *Sicyopterus japonicus* (Perciformes, Gobiidae). Mitochondrial DNA. 2013;24: 191-193. doi: 10.3109/19401736.2012.744980.

20. Chiang TY, Chen IS, Lin HD, Hsiao ST, Ju YM. Complete mitochondrial genome of the amphidromous, red-tailed goby *Sicyopterus lagocephalus* (Pallas) (Teleostei, Gobiidae). Mitochondrial. DNA. 2013 Oct 9. doi: 10.3109/19401736.2013.840600.

21. Maeda K, Mukai T, Tachihara K. A new species of amphidromous goby, *Stiphodon alcedo*, from the Ryukyu archipelago (Gobiidae: Sicydiinae). Cybium. 2011;35: 285-298.

22. Jin X, Gao Y, Xu T, Shi G, Zhao S, Sun Y. Complete mitochondrial genome of the Asian freshwater goby *Synechogobius ommaturus* (Perciformes, Gobioidei). Mitochondrial DNA. 2013;24: 83-85. doi: 10.3109/19401736.2012.717937.

23. Thacker C. Phylogeny of Gobioidei and placement within Acanthomorpha, with a new classification and investigation of diversification and character evolution. Copeia. 2009;2009: 93-104. doi: 10.1643/CI-08-004.

24. Miya M, Friedman M, Satoh TP, Takeshima H, Sado T, Iwasaki W, et al. Evolutionary origin of the Scombridae (tunas and mackerels): members of a Paleogene adaptive radiation with 14 other pelagic fish families. PLOS ONE. 2013;8: e73535. doi: 10.1371/journal.pone.0073535.
